# Resolvin D5, a Lipid Mediator, Inhibits Production of Interleukin-6 and CCL5 Via the ERK-NF-κB Signaling Pathway in Lipopolysaccharide- Stimulated THP-1 Cells

**DOI:** 10.4014/jmb.1907.07033

**Published:** 2019-11-01

**Authors:** Hyun-Woo Chun, Jintak Lee, Thu-Huyen Pham, Jiyon Lee, Jae-Hwan Yoon, Jin Lee, Deok-Kun Oh, Jaewook Oh, Do-Young Yoon

**Affiliations:** 1 Department of Bioscience and Biotechnology, Research Institute of Bioactive-Metabolome Network, Konkuk University, Seoul 05029, Republic of Korea; 2 Department of Stem Cell and Regenerative Biotechnology, Konkuk University, Seoul 0509, Republic of Korea

**Keywords:** Resolvin D5, anti-inflammatory, THP-1 cells, ERK, NF-κB

## Abstract

One of the omega-3 essential fatty acids, docosahexaenoic acid (DHA), is a significant constituent of the cell membrane and the precursor of several potent lipid mediators. These mediators are considered to be important in preventing or treating several diseases. Resolvin D5, an oxidized lipid mediator derived from DHA, has been known to exert anti-inflammatory effects. However, the detailed mechanism underlying these effects has not yet been elucidated in human monocytic THP-1 cells. In the present study, we investigated the effects of resolvin D5 on inflammation-related signaling pathways, including the extracellular signal-regulated kinase (ERK)-nuclear factor (NF)-κB signaling pathway. Resolvin D5 downregulated the production of interleukin (IL)-6 and chemokine (C-C motif) ligand 5 (CCL5). Additionally, these inhibitory effects were found to be modulated by mitogen-activated protein kinase (MAPK) and NF-κB in lipopolysaccharide (LPS)-treated THP-1 cells. Resolvin D5 inhibited the LPS-stimulated phosphorylation of ERK and translocation of p65 and p50 into the nucleus, resulting in the inhibition of IL-6 and CCL5 production. These results revealed that resolvin D5 exerts anti-inflammatory effects in LPS-treated THP-1 cells by regulating the phosphorylation of ERK and nuclear translocation of NF-κB.

## Introduction

Eicosapentaenoic acid (EPA) and docosahexaenoic acid (DHA) are omega-3 (ω-3) essential fatty acids (EFAs) and significant regulators of inflammatory responses [1]. EFAs exert positive effects against inflammatory bowel disease, rheumatoid arthritis, psoriasis, and cancer [2]. Specialized pro-resolvin mediators can be biosynthesized from EPA, yielding resolvins and protectins [3]. Resolvin D5, a lipid mediator derived from DHA via the action of 5-lipooxygenase, is an attractive target for the resolution of inflammation. Similar to other resolvins, resolvin D5 has been expected to possess an anti-inflammatory activity. Systemic treatment with resolvins and protectins protected against colitis and intestinal ischemia/reperfusion-induced inflammation in mice [4]. Moreover, it has been demonstrated that essential fatty acid-derived specialized pro-resolving mediators (SPMs) stimulate the resolution of pain and inflammation. Some resolvins can be used in treatment of chemotherapy-induced peripheral neuropathy (CIPN), a rising health concern in cancer therapies [5]. SPMs have been recognized in inflammation resolution, but specific pro-resolving mediators have conserved structures functioning in host defense, pain, protection of organ and remodeling tissue. Investigations into the pathways of specialized pro-resolving mediators help us in understanding their biological functions [6]. However, due to the limited biological availability of resolvin D5, research regarding its health benefits is still in its early stage [4, 7]. Therefore, to reveal the underlying mechanisms behind the anti-inflammatory effects of resolvin D5, further studies regarding such properties are necessary.

Interleukin (IL)-6 and the chemokine (C-C motif) ligand 5 (CCL5) are significant inflammatory mediators; their expression is upregulated in the initial step of inflammatory responses or tissue damage. However, if produced excessively, these cytokines can aggravate diverse diseases of inflammation, such as rheumatoid arthritis, atherosclerosis, Alzheimer’s disease and acute ischemic stroke [8]. THP-1 cell is the human monocytic leukemia and has been a model for studying the functions and signaling pathways of monocytes/macrophages, and the underlying mechanisms. This cell line is a common model used to evaluate the regulation of monocyte and macrophage activities, which trigger pro-inflammatory cytokine production [9, 10]. THP-1 cells are sensitive to lipopolysaccharide (LPS) and respond by producing many inflammatory cytokines [11]. LPS is a major constituent of the outer wall of gram-negative bacteria and plays a crucial role in regulating inflammation. Immune cells such as monocytes and macrophages can detect LPS and produce certain kinds of pro- and anti-inflammatory cytokines. It has been stated that LPS-stimulated THP-1 cells produce IL-6 and CCL5 by activating the mitogen-activated protein kinase (MAPK) and nuclear factor-kappa B (NF-κB) pathways [11-13]. MAPKs, which are serine/threonine protein kinases, are concerned in essential responses of cells, such as cell differentiation, proliferation, death and survival, and are important for modulating the levels of inflammatory factors. These kinases, such as the extracellular signal-regulated kinases (ERKs), c-Jun NH2-terminal kinases (JNKs), and p38 isoforms, are regulated by phosphorylation cascades and promote the phosphorylation of other proteins, thereby regulating the expression of various genes [14, 15]. NF-κB, a well-known transcription factor and a downstream factor of MAPK pathway, plays a crucial role in immune responses, cellular growth, apoptosis, and inflammation. It is also involved in the transcription of cytokines, enzymes, and adhesion molecules related with chronic diseases of inflammation [16, 17]. NF-κB consists of p65 and p50 subunits as heterodimers. At resting state, NF-κB is present in the cytosol and is bound by IκB, which blocks the nuclear translocation of NF-κB [18]. However, upon external stimulation, NF-κB-bound IκB is phosphorylated and subsequently degraded in the cytosol by several kinases such as MAPK or IκB kinase (IKK). Then, p65 and p50 are translocated into the nucleus where they bind to several sites on their target genes and regulate their expression [19, 20]. It is unexplored whether resolvin D5 would inhibit these MAPKs and the signaling pathways involving these transcription factors. In this study, the anti-inflammatory effects of resolvin D5 were demonstrated in LPS-stimulated human monocytic THP-1 cells; additionally, the relationships between these effects and the MAPK and NF-κB signaling pathways were elucidated.

## Materials and Methods

### Cell Culture

The human monocyte THP-1 cells were purchased from the Korean Cell Line Bank, Korean Cell Line Research Foundation (Korea). The media for culturing THP-1 cells was RPMI 1640 (Welgene Inc., Korea) supplemented with 10% (v/v) heat-inactivated fetal bovine serum (Hyclone Laboratories, USA). The incubation condition is at atmosphere containing 5% CO_2_ at 37°C, and only the cells under 20 passages were used for experiments.

### Raw Materials and Reagents

The polyunsaturated fatty acid (PUFA) standard DHA was obtained from Sigma (USA). To prepare the lipid mediator resolvin D5, the following reaction was performed: 50 mM 4-(2-hydroxyethyl) piperazinyl-1-propanesulfonic acid (EPPS) buffer (pH 8.5) + 100 mg l^-1^ of DHA as a substrate + 14.4 g l^-1^ of recombinant cells, with shaking at 200 rpm for 2 h at 30°C. The reaction solution was extracted with ethyl acetate, and the rotatory evaporator was used for removing the solvent. The collected sample showed >99%purity. After identification by LC-MS/MS, the sample was used as the lipid mediator DHA as recently reported [21].

### MTS Assay

The cell viability was estimated using the 3-(4,5-dimethylthiazol-2-yl)-5-(3-carboxymethoxy phenyl)-2-(4-sulfophenyl)-2H-tetrazolium (MTS) assay. The colorimetric MTS assay is used for evaluating the levels of mitochondrial reductase, which induces the formation of formazan from tetrazolium compound. The THP-1 cells (1 × 10^5^ cells/well) were seeded into 96-well plates; they were then treated with resolvin D5 at non-cytotoxic concentrations (20 to 40 µM) for 24 h along with 1 µg/ml LPS. The cell viability was estimated by the CellTiter 96 AQueous One Solution Assay (Promega, USA) containing MTS and the electron-coupling reagent, phenazine methosulfate. A 20-µl volume of the AQueous One solution was added, followed by 1 h incubation. The samples were evaluated at OD 492 by a microplate reader (Apollo LB 9110, Berthold Technologies GmbH, Germany). The percentage of the cell viability was calculated as the cell viability $(\%)=(\frac{\mathrm{OD}\mathrm{of}\mathrm{treated}}{\mathrm{OD}\mathrm{of}\mathrm{control}})\times 100.$The viability assays were repeated thrice.

### RNA Isolation and Quantitative RT-PCR (RT-qPCR)

An easy-BLUE Total RNA Extraction Kit (iNtRon Biotechnology, Korea) was used for extracting the RNA. The cDNA was transcribed from RNA with M-MuLV reverse transcriptase (New England Biolabs, USA). RT-PCR was performed with a relative quantification protocol using the Roter-Gene 6000 series software 1.7 (Qiagen, Germany) and the Sensi FAST SYBR NO-ROX Kit (BIOLINE, UK). The expression of all the target genes was normalized to that of the housekeeping gene, glyceraldehyde 3-phosphate dehydrogenase (GAPDH). Each sample was run with the following primer sets: IL-6 : TAC CCC CAG GAG AAG ATT CC (forward) and TTT TCT GCC AGT GCC TCT TT (reverse), CCL5 : GCC CAC ATC AAG GAG TAT TTC (forward) and GAG TTG ATG TAC TCC CGA ACC (reverse), GAPDH : TGA TGA CAT CAA GAA GGT GGT (forward) and TCC TTG GAG GCC ATG TAG GCC (reverse).

### Enzyme-Linked Immunosorbent Assay (ELISA)

The THP-1 cells (2 × 10^5^ cells/well) were seeded into 6-well plates and pretreated with resolvin D5 (20 and 40 µM), then were stimulated for 24 h with LPS (1 µg/ml). The levels of IL-6 secretion in the supernatants were estimated using a sandwich ELISA kit (R&D Systems, USA). The 96-well plate was covered with mouse monoclonal anti-human IL-6 and CCL5 antibodies in 4 µg/ml (prepared in phosphate-buffered saline (PBS)) (R&D Systems). The wells were blocked using bovine serum albumin (BSA) in PBS; next, 100 µl of each supernatant was put to each well. The wells were then incubated with biotinylated goat anti-human IL-6 and CCL5 antibodies in 200 ng/ml for 2 h at room temperature. Next, the wells were incubated with streptavidin-conjugated horseradish peroxidase for 20 min, and then NH_2_SO_4_ solution was added to stop the reaction. The color level was analyzed using an Apollo LB 9110 microplate reader at 450 nm (corrected by the absorption at 570 nm). The ELISA analysis was performed in triplicate.

### Western Blotting

The THP-1 cells (2 × 10^5^ cells/well) were pretreated with resolvin D5 (20 and 40 µM) for 1 h; next, these cells were treated with 1 µg/ml of LPS for another 1 h. The proteins were extracted by RIPA buffer (iNtRon Biotechnology) for 2 h at 4°C. Specific primary antibodies used for western blotting include phosphorylated (p-) ERK, JNK, p-38, and GAPDH antibodies (Santa Cruz Biotechnology, USA).

### Cell Fractionation

The THP-1 cells (5 × 10^5^ cells/well) were pretreated with resolvin D5 (20 and 40 µM); next, cells were treated with 1 µg/ml LPS for 1 h. These cells were then harvested and fractionated with the NE-PER nuclear and cytoplasmic extraction reagents (Thermo Fisher Scientific, Inc., USA). Equal amounts of protein were separated by electrophoresis; the resultant bands were transferred onto polyvinylidene difluoride membranes. Specific primary antibodies were used for detecting the cytosolic and nuclear fractions including p65, p50, c-Jun, c-Fos, and signal transducer and activator of transcription 3 (STAT3) (Santa Cruz Biotechnology). Poly (ADP)-ribose polymerase (PARP) (Cell Signaling Technology, USA) and GAPDH (Santa Cruz Biotechnology) were used as markers for the nuclear and cytosolic proteins, respectively.

### Statistical Analysis

The results are presented as the mean ± SD (*n* = 3) and were compared using one-way analysis of variance (ANOVA), followed by Tukey’s HSD test and p-values less than 0.05 were considered statistically significant.

## Results

### Effects of Resolvin D5 on THP-1 Cell Viability

**Fig. 1:**
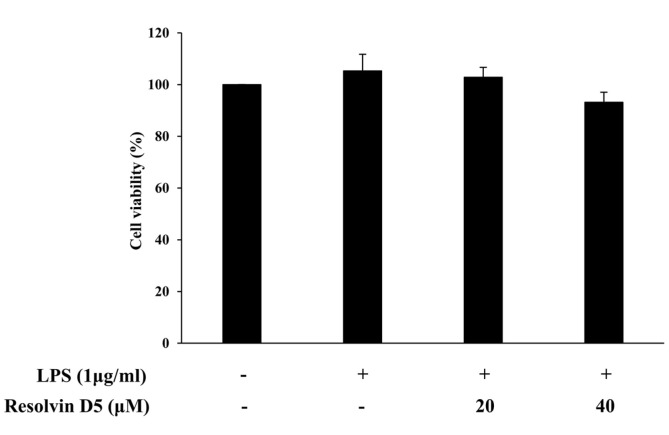
Effects of resolvin D5 on the viability of LPS-stimulated THP-1 cells. The THP-1 cells were pretreated with resolvin D5 for 1 h and then treated with LPS (1 μg/ml) for 24 h. The data were obtained from three independent experiments and reported as the mean ± SD (*n* = 3). *p < 0.05 (LPS alone versus LPS plus resolvin D5), **p < 0.01 (LPS alone versus LPS plus resolvin D5), as calculated by one-way ANOVA.

To assess the non-cytotoxic concentrations of resolvin D5 for THP-1 cells in the treatment or non-treatment of LPS (1 μg/ml) for 24 h, the MTS assay was performed. The results showed that at concentrations of up to 40 μM, resolvin D5 was not cytotoxic towards THP-1 cells (Fig. 1). Thus, resolvin D5 (20–40 μM) was used for all subsequent experiments. EtOH was used at the same concentration as resolvin D5 (40 μM) as a negative control.

### Effects of Resolvin D5 on IL-6 and CCL5 Production in LPS-Treated THP-1 Cells

**Fig. 2:**
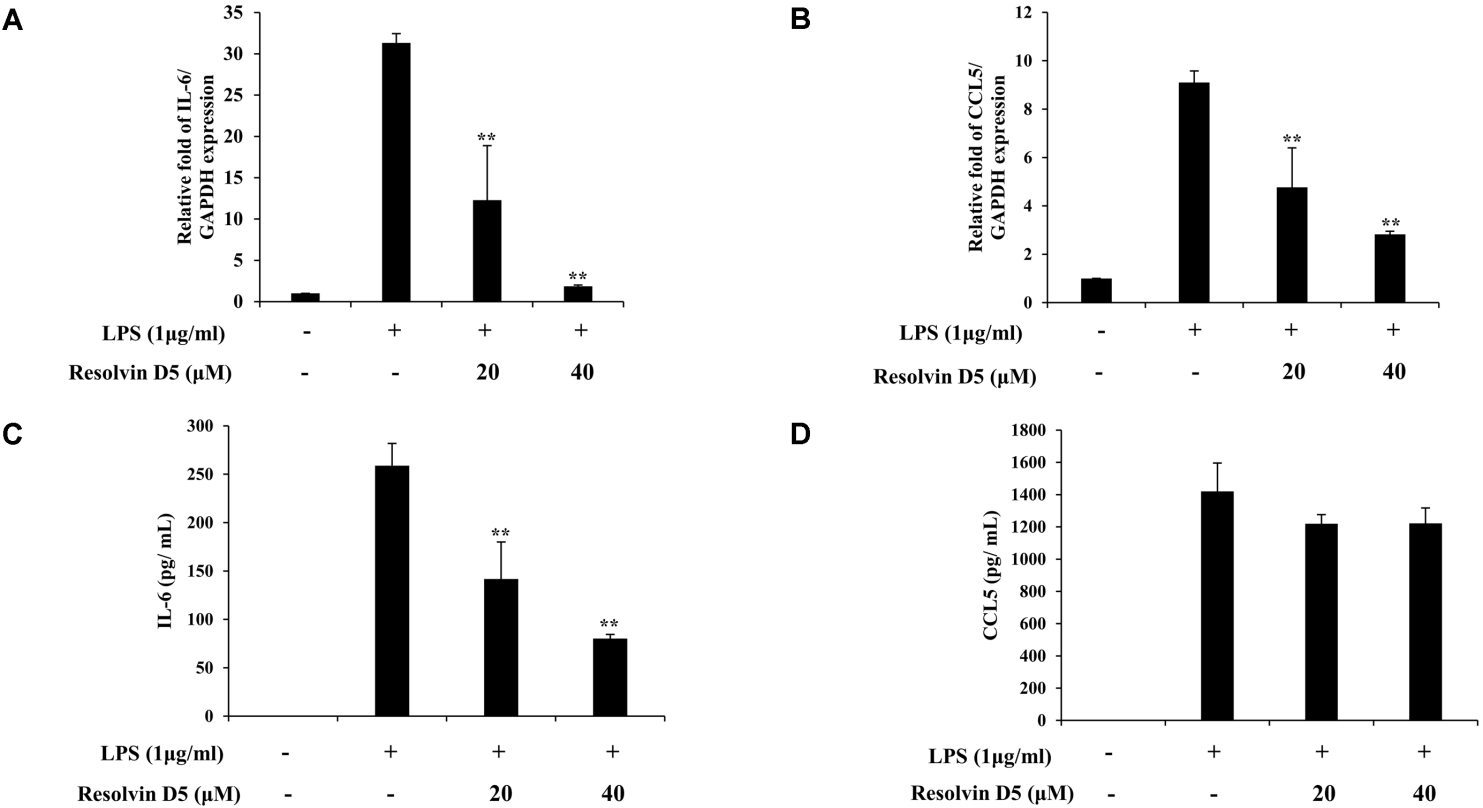
Inhibitory effects of resolvin D5 on LPS-induced IL-6 and CCL5 production in THP-1 cells. THP-1 cells were pretreated with resolvin D5 for 1 h and then treated with LPS (1 μg/ml) for 24 h. The IL-6 and CCL5 mRNA levels were evaluated by RT-qPCR in a resolvin D5 concentration-dependent manner (**A, B**). The IL-6 and CCL5 protein levels were evaluated by ELISA in a resolvin D5 concentration-dependent manner (**C, D**). The data were obtained from three independent experiments and reported as the mean ± SD (*n* = 3). *p < 0.05 (LPS alone versus LPS plus resolvin D5), **p < 0.01 (LPS alone versus LPS plus resolvin D5), as calculated by one-way ANOVA.

 To identify whether resolvin D5 has anti-inflammatory effects in LPS-stimulated THP-1 cells, the cells were pre-treated with resolvin D5 for 1 h, and then with LPS for 24 h. The RT-qPCR analyses revealed that at concentrations of 20–40 μM, resolvin D5 significantly inhibited the IL-6 and CCL5 mRNA levels following LPS treatment (Figs. 2A and 2B). There was no contamination of LPS during resolvin D5 purification because resolvin D5 was extracted from the reaction solution not from the recombinant cells, and resolvin D5 itself did not induce IL-6 or CCL5 mRNA levels (data not shown), suggesting that there was no immunotoxicity in the resolvin D5-treated cells. Furthermore, ELISA results also showed that the secreted protein level of IL-6 in the THP-1 cell supernatants was reduced by resolvin D5. However, unlike the IL-6 level, the secreted CCL5 level was not inhibited notably (Figs. 2C and 2D). Taking all results from Figs. 1 and 2, it was revealed that resolvin D5 neither exerted cytotoxicity nor immunotoxicity and suppressed the expression or production of pro-inflammatory cytokine IL-6 and chemokine CCL5.

### Effects of Resolvin D5 on MAPK Signaling in LPS-Treated THP-1 Cells

**Fig. 3:**
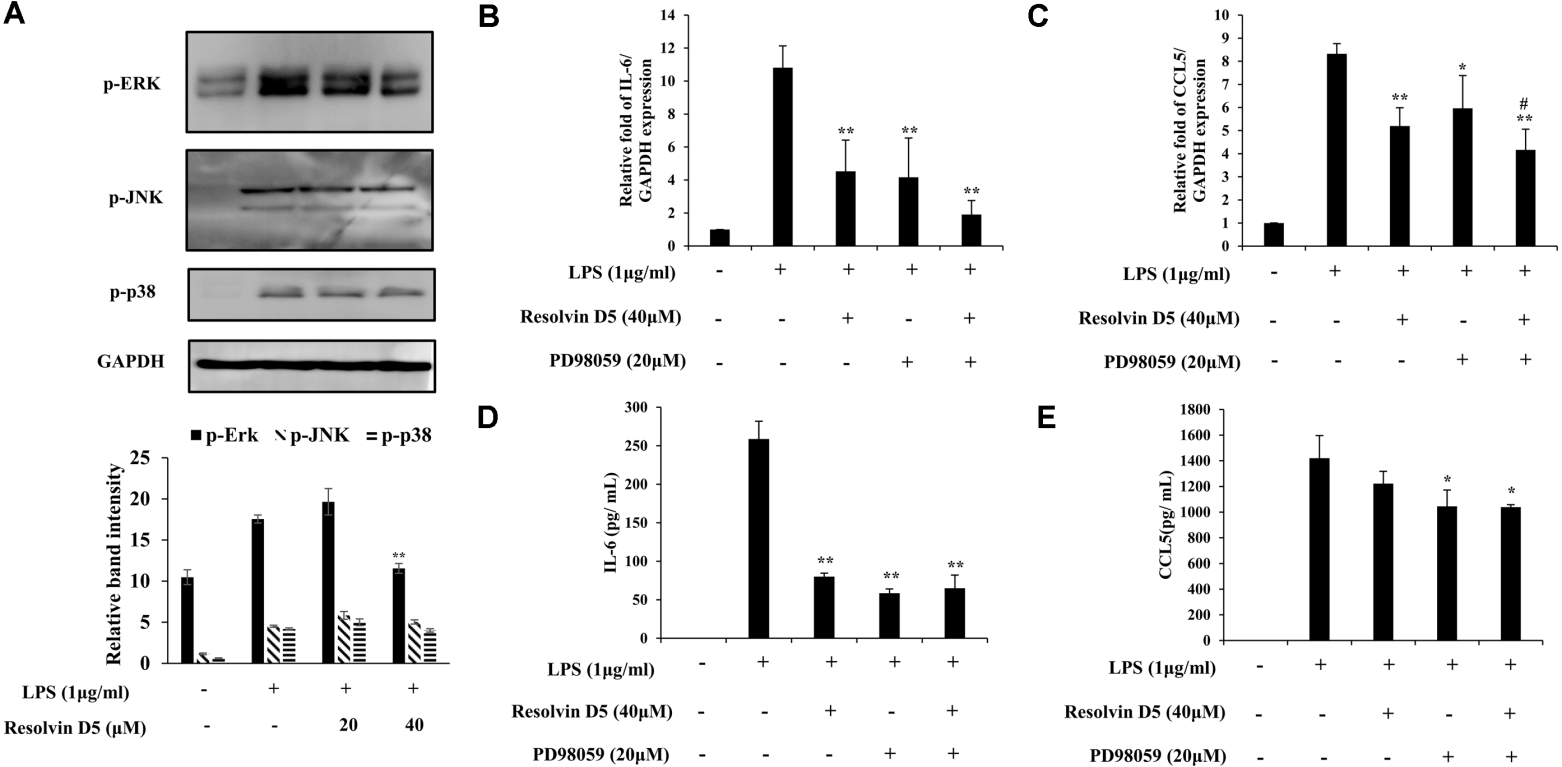
Effects of resolvin D5 on LPS-induced phosphorylation of MAPKs. The phosphorylation of MAPKs was evaluated by western blotting in a resolvin D5 dose-dependent manner (**A**). The THP-1 cells were pretreated with resolvin D5 (40 μM) with or without PD98059 (20 μM) for 1 h and then treated with LPS (1 μg/ml) for 24 h. The mRNA and protein levels were determined by RT-qPCR (**B, C**) and ELISA (**D, E**), respectively. The data were obtained from three independent experiments and reported as the mean ± SD (*n* = 3). *p < 0.05 (LPS alone versus LPS plus resolvin D5), **p < 0.01 (LPS alone versus LPS plus resolvin D5), ^#^ *p* < 0.05 (LPS and ERK inhibitor PD98059 versus LPS and ERK inhibitor PD98059 plus resolvin D5), ^##^ *p* < 0.01 (LPS and ERK inhibitor PD98059 versus LPS and ERK inhibitor PD98059 plus resolvin D5), as calculated by one-way ANOVA.

To identify whether resolvin D5 alters the LPS stimulation of MAPK signaling, several key MAPK members were examined including ERK, JNK, and p38. The phosphorylation of ERK was attenuated by resolvin D5 dose-dependently in THP-1 cells, while that of JNK and p38 was not altered (Fig. 3A). Subsequently, the production of CCL5 and IL-6 was evaluated after the treatment with the ERK inhibitor, PD98059, to determine whether CCL5 and IL-6 expressions were modulated via ERK. Pre-treatment with PD98059 (20 μM) for 1 h before LPS treatment in the absence or presence of resolvin D5 (40 μM) attenuated the production of CCL5 and IL-6. These data indicated that LPS-induced CCL5 and IL-6 secretion was reduced by resolvin D5 via inhibition of the ERK/MAPK signaling pathway (Figs. 3B–3E).

### Effects of Resolvin D5 on Transcription Factor Activities

**Fig. 4:**
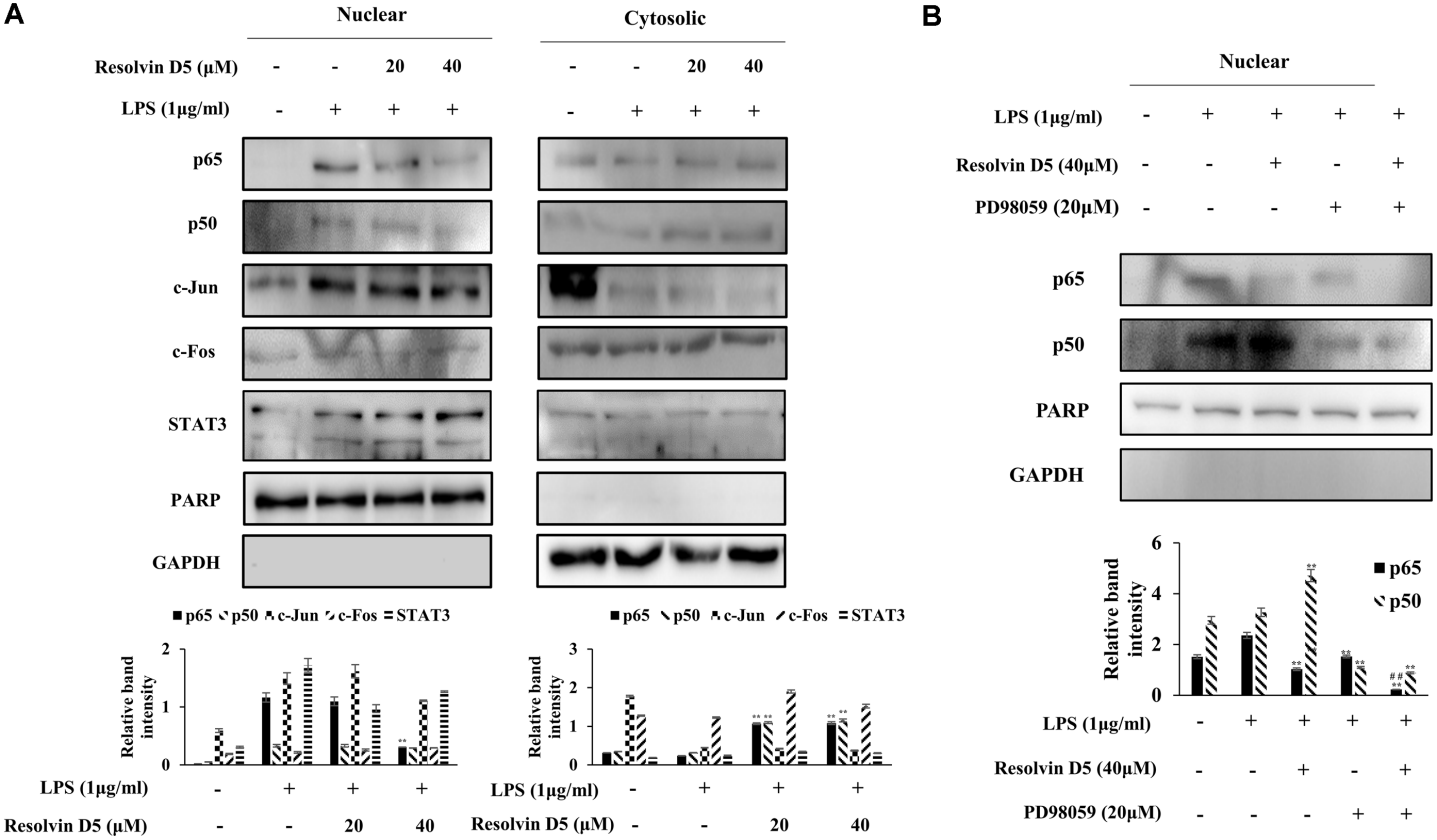
Effects of resolvin D5 on the translocation of transcription factors into the nucleus in LPS-stimulated THP-1 cells. The cells were pretreated with resolvin D5 (20 or 40 μM) alone (**A**) and resolvin D5 (20 or 40 μM) + PD98059 (20 μM) (**B**) for 1 h; they were then stimulated with LPS (1 μg/ml) for 1 h. The translocation of NF-κB, AP-1, and STAT3 was evaluated by the western blotting analysis of the cytosolic and nuclear extracts of the cells. The data were obtained from three independent experiments and reported as the mean ± SD (*n* = 3). *p < 0.05 (LPS alone versus LPS plus resolvin D5), **p < 0.01 (LPS alone versus LPS plus resolvin D5), ^#^ *p* < 0.05 (LPS and ERK inhibitor PD98059 versus LPS and ERK inhibitor PD98059 plus resolvin D5), ^##^ *p* < 0.01 (LPS and ERK inhibitor PD98059 versus LPS and ERK inhibitor PD98059 plus resolvin D5), as calculated by one-way ANOVA.

Transcription factors are involved in the regulation of the expression of various genes via their phosphorylation and translocation. Therefore, the translocation of several transcription factors from the cytosol to the nucleus was investigated. NF-κB is a well-known transcription factor; the nuclear fractionation results revealed that the LPS-induced translocation of NF-κB was decreased by resolvin D5 (Fig. 4A).

Furthermore, to identify the relationship between NF-κB and the ERK signaling pathway, nuclear fractionation of the LPS-treated THP-1 cells was performed in the absence or presence of PD98059. As expected, the nuclear translocation of NF-κB was attenuated by PD98059 and resolvin D5 (Fig. 4B). To investigate the effects of Resolvin D5 on the transcriptional activity of NF-κB, the plasmid vectors containing NF-κB promoter were transfected into cells, and the luciferase assay was performed. Afterwards, the cells were pre-treated with resolvin D5 (20 or 40 μM) for 1 h followed by LPS (1 μg/ml) for another 1 h. The results revealed that the transcriptional activity of NF-κB was significantly inhibited by resolvin D5 in LPS treated cells (Fig. S1). These results demonstrated that the anti-inflammatory effects of resolvin D5 involved the regulation of ERK and NF-κB.

## Discussion

Inflammatory responses ultimately result in the elimination of infectious agents and promote wound healing following tissue injuries. Under normal conditions, these inflammatory responses are well regulated to prevent damage to the host. However, in case of abnormal inflammatory responses, the production of inflammatory mediators is not properly regulated; this leads to chronic inflammatory diseases [22, 23], which can be promoted via synthesis of certain pro-inflammatory cytokines such as IL-6, and chemokines such as CCL5 [24, 25].

IL-6 is produced at the site of inflammation and plays a crucial role in the acute inflammation process, as defined by a variety of biological and clinical features, such as production of acute-phase proteins. The combination of IL-6 and its soluble receptor sIL-6Rα determines the transition from acute to chronic inflammation by alerting the nature of immune cell infiltration [26]. Furthermore, IL-6 is a significant mediator of inflammation, and strongly induces the production of different acute-phase proteins such as C-reactive protein (CRP), a commonly used marker in clinical laboratories to evaluate the severity of inflammation. It is also important in local inflammation by stimulating the differentiation of CD4-positive T cells into effector B cells and T cells into immunoglobulin-producing cells, and inducing IL-8 production, leading to the recruitment of leukocytes to the inflamed lesion [26-28]. The chemokine CCL5 is included in the C-C chemokine family; the other members of this family are CCL3 and CCL4. CCL5 is derived from macrophages, synovial fibroblasts, T lymphocytes and certain types of tumor cells, and plays crucial roles in the recruitment of certain leukocytes such as T cells, macrophages, and polymorphic nuclear cells, to inflammatory sites. In collaboration with specific cytokines that are produced by T cells, such as IL-2 and IFN-γ, CCL5 also stimulates the activation and proliferation of certain natural killer cells to activate C-C chemokine-activated killer cells [29, 30]. Infection and tissue damage result in release of leukotrienes and prostaglandins as pro-inflammatory lipid mediators, which can cause inflammation and stimulate the formation of neutrophils in the blood. The bioactive local mediators, resolvins which required enzymatic generation from the omega-3 essential fatty acid EPA were first identified in resolving inflammatory exudates in vivo and carried potent stereoselective biological actions [31]. For treating osteopenia and osteoporosis, SPMs offer a significant improvement in safety and patient comfort. Similarly, it has been reported that lipoxin (LX), an endogenous SPMs, reduced infantile eczema showing no apparent toxicity or side effects [32]. Neutrophils differentiate into macrophages via monocytes. These macrophages then generate resolvin D5, which resolves inflammation and infection and has low cell toxicity in the human body [32, 33]. In the present study, the secretion levels of CCL5 and IL-6 increased drastically in LPS-stimulated THP-1 cells. However, RT-qPCR and ELISA analyses revealed the inhibitory effects of resolvin D5 treatment on the protein and mRNA expression levels of CCL5 and IL-6 respectively (Fig. 2).

To further elucidate the mechanisms how resolvin D5 inhibits cytokine production, the activation of MAPKs (ERK, JNK, and p38), which are known to be associated with the modulation of these cytokines, was examined. MAPKs comprise a family of serine/threonine protein kinases that are involved in regulating important cellular processes such as cellular stress, cell proliferation, survival and differentiation, and inflammatory responses [34]. It was observed that ERK, JNK, and p38 phosphorylation were enhanced after LPS treatment. However, it was also revealed that resolvin D5 attenuated inflammatory responses by inhibiting ERK phosphorylation, but not JNK and p38 phosphorylation (Fig. 3A). Furthermore, IL-6 and CCL5 production following treatment with PD98059 was examined to determine if the effects of resolvin D5 on their production would be regulated via ERK (Figs. 3B–3E).

The ubiquitous transcription factor NF-κB is important in inflammation, and responsible for the modulation of genes encoding proinflammatory cytokines such as TNF-α, IL-1, IL-6, IL-12, inducible enzymes such as cyclooxygenase (COX)-2, and inducible nitric oxide synthase (iNOS) [35, 36]. It is activated via signaling cascades triggered by extracellular stimuli such as LPS. Cell culture studies have shown that the ω-3 fatty acids EPA and DHA inhibit the LPS-induced production of TNF-α, IL-1, IL-6, COX-2, and iNOS in monocytes and macrophages. Additionally, ω-3 fatty acids inhibit the LPS-induced activation of NF-κB [37-40]. Resolvin D5, a lipid mediator that is biosynthesized from DHA, also inhibited the expression of inflammatory mediators via the NF-κB pathway in LPS-stimulated THP-1 cells. The nuclear translocation of the NF-κB subunits, p65 and p50, were upregulated after LPS stimulation; however, resolvin D5 treatment attenuated this translocation (Fig. 4A). Other transcription factors involved in inflammation, such as activated protein-1 (AP-1) and STAT3, were also examined; resolvin D5 did not affect the translocation of these transcription factors. To determine the relationship between NF-κB and ERK, LPS-treated THP-1 cells were treated with the ERK inhibitor PD98059 in the presence or absence of resolvin D5. Indeed, the translocation of p65 was inhibited by PD98059, and it was revealed that NF-κB was regulated via the ERK signaling pathway (Fig. 4B). To confirm the effects of resolvin D5 on NF-κB transcriptional activation, a transient transfection and luciferase assay were performed. The results revealed an effective inhibitory effect of resolvin D5 on the transcriptional level of NF-κB after LPS stimulation (Fig. S1).

Collectively, our results showed that resolvin D5 downregulated the ERK phosphorylation and the nuclear translocation of p65 and p50 (Fig. 4A), resulting in the inhibition of IL-6 and CCL5 expression and/or production (Fig. 2). Therefore, resolvin D5 was ascertained to be a potential anti-inflammatory compound.

## Supplemental Materials



Supplementary data for this paper are available on-line only at http://jmb.or.kr.
