# Supplementary material for: Resolvin D5, a Lipid Mediator, Inhibits Production of Interleukin-6 and CCL5 Via the ERK-NF-κB Signaling Pathway in Lipopolysaccharide- Stimulated THP-1 Cells
Source: J Microbiol Biotechnol. 2019 Nov 1;30(1):85–92. doi: 10.4014/jmb.1907.07033 (PMC9728331; doi:10.4014/jmb.1907.07033)
Supplement: Supplementary file 1 [file JMB-30-1-85-supple.pdf]

J. Microbiol. Biotechnol.
https://doi.org/10.4014/jmb.1907.07033

jmb

MATERIALS AND METHODS

S1. Transient transfection and luciferase assay

Cells were seeded in 6-well plates at a density of 10^6 cells/ml and then transiently co-transfected with the pGL34xNF-κB promoter (1 μg/ml) and pRL-null Renilla luciferase plasmid (0.1 μg/ml) using Lipofectamine 2000 (Invitrogen, CA, USA). After 24 h of incubation, cells were pretreated with resolvin D5 (20 or 40 μM) for 1 h and then LPS (1μg/ml) for 24 h. Cells were disrupted and protein extraction was performed using the Luciferase Reporter Assay System (Promega, WI, USA). The firefly luciferase activities were measured by VICTOR X3 (PerkinElmer Inc., MA, USA), and normalized to the Renilla luciferase activities.

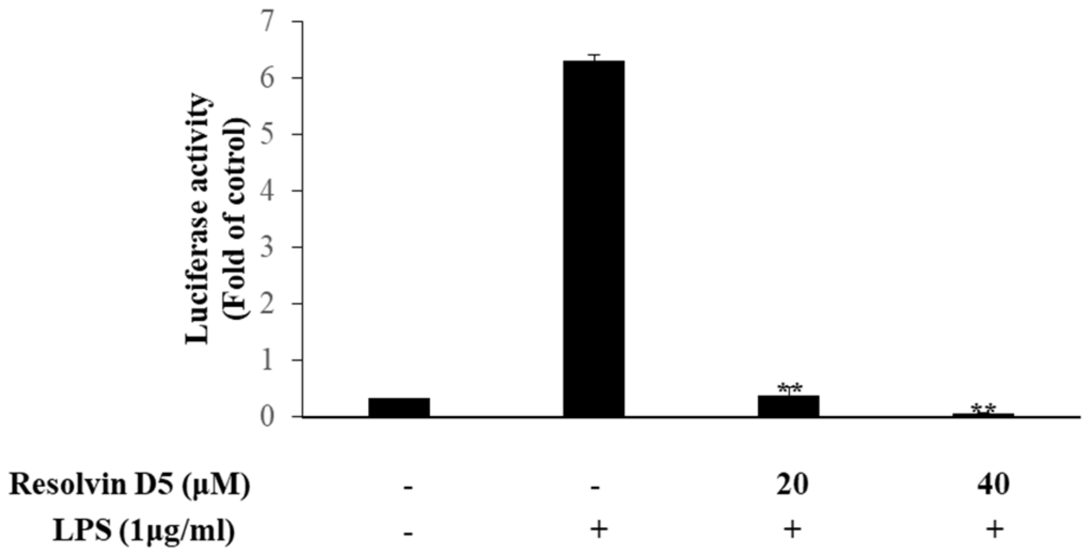

Fig. S1 The luciferase activities of NF-κB were measured and normalized to Renilla luciferase signals. The data were obtained from three independent experiments and reported as the mean ± SD (n = 3). **$P < 0.01$ (LPS alone versus LPS plus resolvin D5), as calculated by one-way ANOVA.
